# Correlations between H_2_ Permeation and Physical/Mechanical Properties in Ethylene Propylene Diene Monomer Polymers Blended with Carbon Black and Silica Fillers

**DOI:** 10.3390/ijms24032865

**Published:** 2023-02-02

**Authors:** Jae K. Jung, Ji H. Lee, Sang K. Jeon, Nae H. Tak, Nak K. Chung, Un B. Baek, Si H. Lee, Chang H. Lee, Myung C. Choi, Hyun M. Kang, Jong W. Bae, Won J. Moon

**Affiliations:** 1 Korea Research Institute of Standards and Science, Hydrogen Energy Materials Research Center, Daejeon 34113, Republic of Korea; 2 Department of Biochemical and Polymer Engineering, Chosun University, Gwangju 61452, Republic of Korea; 3 Rubber Research Division, Korea Institute of Footwear & Leather Technology, Busan 47154, Republic of Korea; 4 Gwangju Center, Korea Basic Science Institute, Gwangju 61186, Republic of Korea

**Keywords:** carbon black, silica, H_2_ uptake, diffusion, permeation, density, correlation

## Abstract

H_2_ permeation in peroxide-crosslinked EPDM blended with carbon black (CB) and silica fillers was studied at pressures ranging from 1.2 MPa to 90 MPa via the volumetric analysis technique. H_2_ uptake in the CB-filled EPDM revealed dual-sorption behaviors via Henry’s law and the Langmuir model, which were attributed to H_2_ absorption by the polymer chains and H_2_ adsorption at the filler interfaces, respectively. Additionally, single-sorption mechanisms were observed for neat EPDM and silica-blended EPDM according to Henry’s law, indicating H_2_ absorption by the polymer chain. The linear decreases in the diffusivity with filler content for the silica-blended EPDMs were attributed to increases in the diffusion paths caused by the filler. Exponential decreases in the diffusivity with increasing filler content and in the permeation with the physical/mechanical properties for CB-filled EPDMs were caused by decreases in the fractional free volume due to increased densities for the EPDM composites. Moreover, good filler-dependent correlations between permeability and density, hardness, and tensile strength were demonstrated for EPDMs used as sealing materials for O-rings. From the resulting equation, we predicted the permeation value without further measurements. Thus, we can select EPDM candidates satisfying the permeation guidelines used in hydrogen infrastructure for the future hydrogen economy.

## 1. Introduction

The infrastructure used for accelerating the hydrogen economy causes an increase in the number of materials or components coming into contact with high-pressure hydrogen. In particular, polymers are widely used in sealing components for hydrogen environments and are directly exposed to high-pressure H_2_ in service environments [1,2,3,4]. O-ring seals, gaskets, control valves, connectors, and nonmetallic pipelines are the primary applications for the polymer materials [1,5,6,7,8,9].

H_2_ quickly penetrates into the polymer membrane up to its equilibrium level. When the high pressure is released into the atmosphere, the rapid depressurization causes supersaturation of the membrane [10,11]. When the gas is released faster than it diffuses, it is transported out of the polymer and exhibits bubble nucleation, which causes irreversible damage to the polymer, such as microcracking, swelling, fracture, and embrittlement. This ultimately causes sealing failure in the O-rings used in hydrogen refueling stations (HRSs) and hydrogen fuel cell vehicles (HFCVs) and results in gas leakage [10].

In addition, leakage due to damage of the seal and H_2_ gas leakage through the O-ring under high pressure can be generated in two different situations [11]: one involves gap leakage between the seal and the groove due to insufficient contact, and the other involves gas permeation through the sealing polymer. These leaks should be measured by determining the permeation of the base material or O-ring, employing the appropriate techniques.

The international standard for the type 4 tanks used in HFCVs specifies that H_2_ permeation through the leakage must be less than 46 Ncm^3^ dh^−1^m^−3^ at 70 MPa and 55 °C [12]. ISO19880-5 requires the amount of H_2_ permeation from the dispensing hose to be less than 500 Ncm^3^ h^−1^ m^−1^ at 87.5 MPa [13]. If we design hydrogen apparatuses such as H_2_ vessels and control valves, appropriate sealing materials for O-rings should also be selected based on the hydrogen permeability database for polymers determined during practical use under high pressures. Thus, H_2_ permeation, diffusivity, and solubility are the dominant parameters used for designing or selecting sealing materials suitable for use [14]. For this reason, effective, precise, and traceable techniques for measuring hydrogen permeation parameters are absolutely necessary.

Meanwhile, resistive polymeric rubbers have been developed to preclude hydrogen embrittlement. Rubber matrix compounds are varied with reinforcing fillers, such as carbon black (CB), silica, crosslinking agents (sulfur or peroxide), plasticizers, and activators. Various rubber matrices used for addition of the fillers, crosslinking agents, plasticizers, and activators were fabricated; these included acrylonitrile butadiene rubber (NBR), ethylene propylene diene terpolymer (EPDM), and fluoroelastomer (FKM). In particular, EPDM exhibits excellent heat and chemical resistance and low-temperature properties, flexibility over a wide temperature range, and H_2_ barrier properties [15,16,17]. EPDM is the most widely utilized material for seals, tubes, and gaskets in H_2_ infrastructure, such as HFCVs and HRSs. Therefore, EPDM rubbers reinforced with CB exhibiting different particle sizes, silica, and sulfur/peroxide crosslinking agents are candidates for the O-ring materials used in gas seals, which experience high pressures up to 90 MPa in HRSs [18].

This research is focused on the H_2_ permeation properties of sulfur/peroxide crosslinked EPDM containing CB and silica fillers. By investigating the effects of filler loadings on the H_2_ gas permeabilities in these composites, we clarify the sorption and diffusion phenomena and filler-induced permeation of the polymer. The H_2_ permeation characteristics of the polymer were precisely measured using volumetric analyses and a diffusion analysis program [19,20]. The H_2_ uptake, diffusion coefficient, and permeability of the EPDM composites filled with three fillers were investigated to study the effects of exposure pressure, filler content, and filler type.

Furthermore, permeation is associated with the physical and chemical properties of polymers [21,22,23,24]. Many studies have been conducted to clarify the correlation mechanisms and relate them to the appropriate theories. Specifically, permeation is dependent on the free volume of the polymer [25,26,27,28,29,30,31,32,33], which is mainly affected by physical/chemical properties such as the glass/rubber phases, crystallinity, amorphous volume fractions, crosslinking density, and density. Furthermore, mechanical properties such as tensile strength and hardness could be correlated with gas permeability. The primary aim of this study is to seek correlation functions relating permeation to investigated fundamental properties. On the basis of these correlations, we can predict the permeation parameters of rubbery polymers comprising filled EPDM composites without additional experiments.

## 2. Results and Discussion

### 2.1. Transmission Electron Microscopy

**Figure 1:**
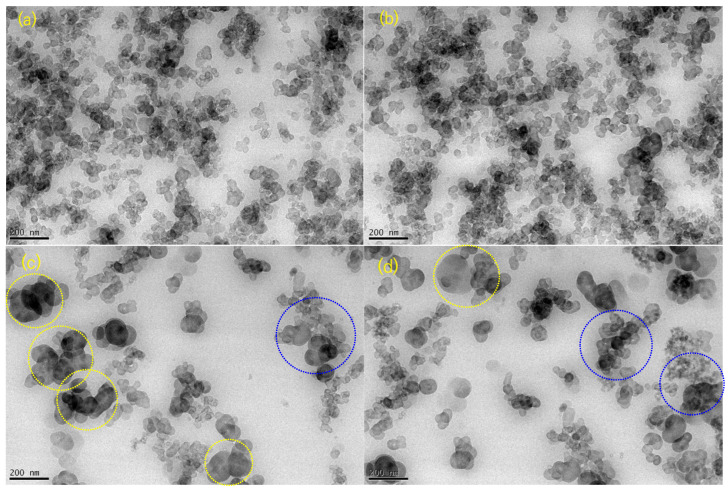
TEM images showing the CB particles (black or gray spherical shape) and aggregates in the peroxide-crosslinked CB-filled EPDM composite with (**a**,**b**) HAF20 and (**c**,**d**) SRF20: (**a**,**b**) are images taken for different parts of the EPDM HAF20 specimen; (**c**,**d**) are images taken for different parts of the EPDM SRF20 specimen.

The method and sample preparation used in obtaining transmission electron microscopy (TEM) images are described elsewhere [20]. Figure 1a–d show the TEM micrographs of EPDM HAF20 and SRF20. The homogenous distribution of CB as a form of black or gray spherical shape in the rubber matrix is shown in Figure 1a,b. As shown in Figure 1c,d, large agglomerates (indicated with yellow dotted circles) of SRF CB were observed in the EPDM SRF20 composite. The shapes and distributions of the CB filler were identified from the TEM image showing the CB filler particles in the rubber matrix. EPDM HAF20 and SRF20 had spherical shapes with the corresponding polarized particle sizes. In particular, the particles were distributed as partially dense aggregates, as shown in Figure 1c,d, indicated with blue dotted circles.

Well-dispersed fillers with small particle sizes, such as the HAF CB filler, cause large filler surface areas and strong interactions between the polymer and the filler, thus affecting the permeation properties. In the HAF CB-filled EPDM polymer crosslinked with peroxide, the degree of filler dispersion was measured according to the testing method (ASTM D7723 and ISO 11345). The measured dispersion degrees for the EPDM rubber composites blended with HAF CB and SRF CB fillers are shown in Table 1. The dispersions for EPDM HAF20 and SRF20 were found to have low dispersion degrees of 88∼90%. However, the dispersions for EPDM HAF40, HAF60, SRF40, and SRF60 were found to exhibit dispersion degrees of 97∼99%, which indicated well-dispersed fillers in the rubber network. To date, we have not found an appreciable correlation between permeation and dispersion degree for EPDM composites.

### 2.2. Measurement Method and Diffusion Analysis

For the volumetric measurements, we utilized graduated cylinders in which the H_2_ emitted by the specimen was collected and measured. After exposure in the high-pressure chamber and subsequent decompression, the samples were loaded into their corresponding gas-cell spaces at the tops of the graduated cylinders. Details of the method are described elsewhere [19,20].

The number of moles (∆n) of H_2_ released into the graduated cylinder was converted to a concentration [*C*(t)] per mass for the H_2_ released from the rubber sample:

(1) $$C\left(t\right)\left[\mathrm{wtppm}\right]=∆n\left[\mathrm{mol}\right]\times \frac{m_{H2}\;\left[\frac{g}{\mathrm{mol}}\right]}{m_{sample}\left[g\right]}\times 10^{6}$$

where $m_{H2}$ [g/mol] is the H_2_ molar mass, equal to 2.016 g/mol, and $m_{sample}$ is the sample mass.

Assuming that H_2_ desorption is a Fickian diffusion process, the concentration $C_{E}\left(t\right)$ of the emitted H_2_ was computed as [34,35]

$$C_{E}\left(t\right)/C_{\infty }=1-\frac{32}{\pi^{2}}\times \left[\sum_{n=0}^{\infty }\frac{\exp\left\{\frac{-{\left(2n+1\right)}^{2}\pi^{2}Dt}{l^{2}}\right\}}{{\left(2n+1\right)}^{2}}\right]\times \left[\sum_{n=1}^{\infty }\frac{\exp\left\{-\frac{D\beta_{n}^{2}t}{\rho^{2}}\right\}}{\beta_{n}^{2}}\right]$$

$$=1-\frac{32}{\pi^{2}}\times \left[\frac{\exp\left(-\frac{\pi^{2}Dt}{l^{2}}\right)}{1^{2}}+\frac{\exp\left(-\frac{3^{2}\pi^{2}Dt}{l^{2}}\right)}{3^{2}}+\ldots ,+\frac{\exp\left(-\frac{{\left(2n+1\right)}^{2}\pi^{2}Dt}{l^{2}}\right)}{{\left(2n+1\right)}^{2}}+\ldots ,\right]$$

(2) $$\times \left[\frac{\exp\left(-\frac{D\beta_{1}^{2}t}{\rho^{2}}\right)}{\beta_{1}^{2}}+\frac{\exp\left(-\frac{D\beta_{2}^{2}t}{\rho^{2}}\right)}{\beta_{2}^{2}}+\ldots ,+\frac{\exp\left(-\frac{D\beta_{n}^{2}t}{\rho^{2}}\right)}{\beta_{n}^{2}}+\ldots ,\right]$$

where $\beta_{n}$ is the root of the zero-order Bessel function *J_0_(β_n_)* with *β*_1_ = 2.40483, *β*_2_ = 5.52008, *β*_3_ = 8.65373, …, *β*_50_ = 156.295. Equation (2) is an infinite series expansion with two summations. It gives the solution to Fick’s second diffusion equation for a cylindrical sample. Here, *C_E_* = 0 at *t* = 0 and *C_E_* =$\;C_{\infty }$ at *t* = ∞. $C_{\infty }$ is the saturated H_2_ concentration at infinite time, i.e., the H_2_ uptake. *D* is the diffusion coefficient. ρ  and l are the radius and thickness of the cylindrical specimen, respectively. The *D* and $C_{\infty }$ values for the EPDM composites were determined with a diffusion analysis program. The application example for the diffusion analysis program and the procedure are described in the literature [20].

### 2.3. Filler Effects on H_2_ Uptake

**Figure 2:**
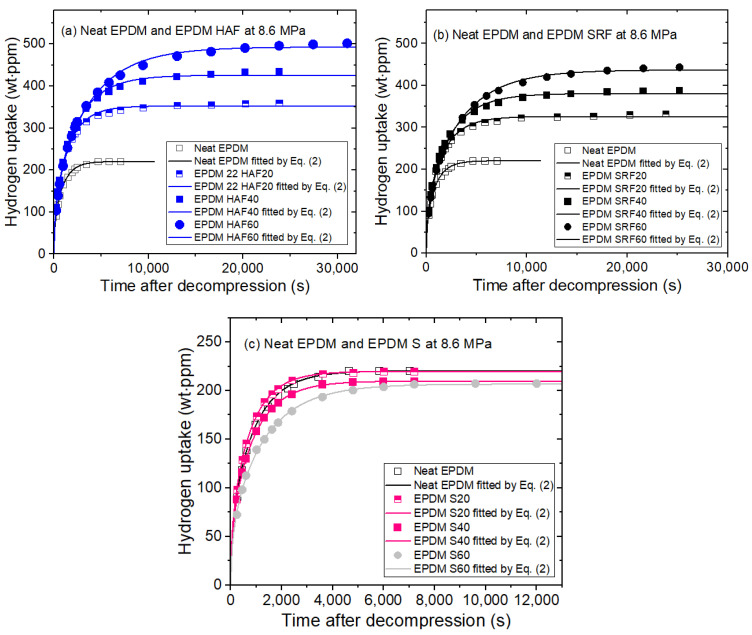
H_2_ uptake by the (**a**) EPDM HAF series, (**b**) EPDM SRF series, and (**c**) EPDM S series after hydrogen exposure at 8.6 MPa. The solid lines are the least-squares fits to Equation (2) using the diffusion analysis program. The results for neat EPDM are included in the three panels for comparison with those for the blended EPDM composites with fillers. The squared correlation coefficient (R^2^ = 0.99) indicated good fits between the data and Equation (2).

The time-dependent H_2_ emission after decompression from pressures ranging from 1.2 to 90 MPa at 23 °C was measured for ten EPDM composites, including neat EPDM and samples blended with CB or silica. Figure 2 shows plots of the H_2_ uptake versus elapsed time for the 10 EPDM rubbers after hydrogen exposure at 8.6 MPa. The primary trend showed greater H_2_ uptake by the CB-filled EPDM composites compared with neat EPDM. This was attributed to H_2_ adsorption resulting from the presence of the CB filler. The H_2_ content in the CB-filled EPDM composites increased with increasing CB content. The filler effects on the SRF CB-filled EPDM composites were similar to those of the HAF CB-filled EPDM samples. The minute increase seen in H_2_ uptake for the HAF CB-filled EPDM might have been caused by the larger specific surface area of the HAF CB filler relative to that of the SRF CB filler. In the silica-filled EPDM composites, the H_2_ uptakes seen with silica filler content were not detectably different from that seen for the neat EPDM polymer. This implied that H_2_ was not adsorbed onto the silica surface, as it was with the CB filler.

**Figure 3:**
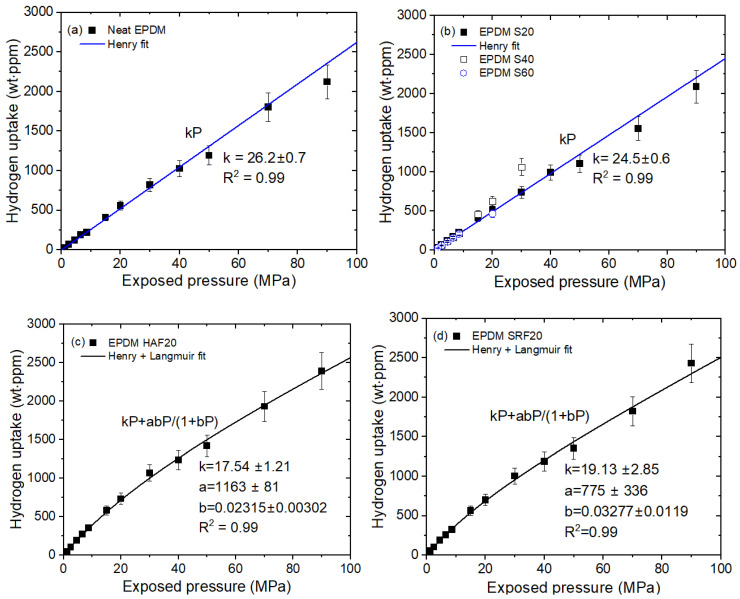
Relationship between H_2_ uptake ($C_{\infty }$) and exposure pressure for (**a**) neat EPDM, (**b**) the EPDM S series, (**c**) EPDM HAF20, and (**d**) EPDM SRF20. The blue and black lines show the fits to the Henry model and the dual-mode (Henry and Langmuir) model, respectively. The legends show the least-squares fit equations and the correlation coefficient R^2^.

We measured the H_2_ emission contents versus exposure pressures for the nine EPDM composites blended with fillers and the neat EPDM. Figure 3 shows representative H_2_ uptake data for the four EPDM composites as a function of pressure. Figure 3a–d show the pressure-dependent H_2_ uptake data for neat EPDM, EPDM composites with the silica filler, EPDM HAF20, and EPDM SRF20, respectively.

The H_2_ uptakes (*C*_∞_) for neat EPDM (Figure 3a) and EPDM S20 (Figure 3b) were proportional to the applied pressure up to 90 MPa and thereby obeyed Henry’s law [36,37]. This behavior was responsible for the absorption of H_2_ into the polymer matrix. However, as shown in Figure 3c,d, the hydrogen uptake for EPDM HAF20 and SRF20 deviated from Henry’s law at pressures above 15 MPa; this was caused by H_2_ adsorbed onto the surface of the CB filler. Thus, dual sorption was observed for all CB-blended EPDM composites. The dual-mode sorption behavior for the pressure range up to 90 MPa is expressed as follows:

(3) $$C_{\infty }=kP+\frac{abP}{1+bP}$$

where $C_{\infty }$ is total H_2_ uptake. The first term indicates Henry’s law, with a Henry’s law coefficient of *k*. The second term indicates the Langmuir model [38,39], where a is the maximum adsorption quantity (or capacity parameter) and b is the adsorption equilibrium constant (or Langmuir hole affinity parameter). The fitting results for H_2_ uptake according to Equation (3) are summarized in Table 2.

**Figure 4:**
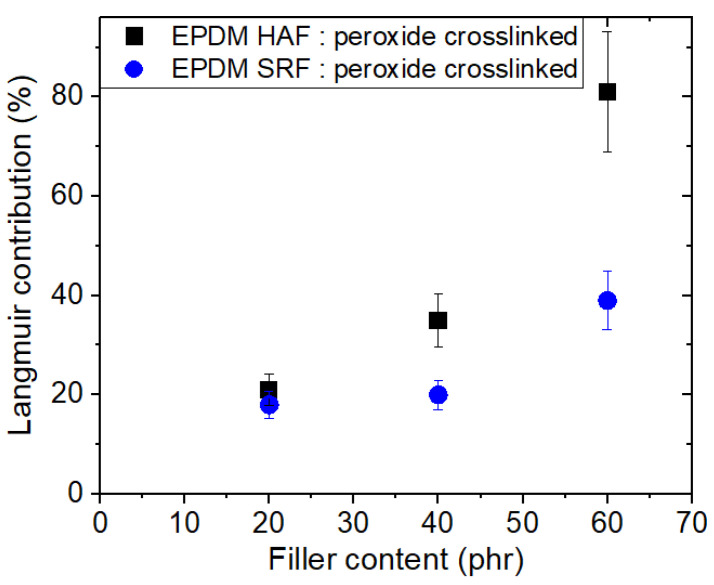
Langmuir contribution versus filler content for the CB-filled EPDM composites.

The Langmuir contribution implied that the quantity of H_2_ adsorbed increases with increasing filler content, as shown in Figure 4. The deviations at 60 phr CB in the filled EPDM composites indicated an abrupt increase in H_2_ adsorption, which may be attributed to the formation of the H_2_ channels that leads to percolation effects with many fillers.

Langmuir sorption is applied to porous solids in a gas–polymer system. The Langmuir sorption sites in a system are the holes or voids that arise from the nonequilibrium states of glassy polymers. A gas sorption isotherm for a glassy polymer occurs below the glass transition temperature (T_g_) and is dependent on the pressure. This behavior is characteristic of dual-mode sorption composed of Henry’s law absorption occurring in an equilibrium state and Langmuir adsorption occurring in a nonequilibrium state [40]. The nonequilibrium state is associated with the extra free volumes or unrelaxed free volumes of glassy polymers [41]. The validity of the dual-mode assumption was confirmed [42]. Therefore, the dual-sorption model is an effective tool for understanding sorption by glassy polymers.

It was previously demonstrated for HAF CB-blended NBR [43] that investigations of the rubbery polymer above T_g_ revealed dual-sorption behavior due to the presence of a porous HAF CB filler. H_2_ molecules could be absorbed by the rubbery NBR and simultaneously adsorbed by the porous filler, leading to dual-mode sorption similar to that seen for the glassy phase of the polymer. Thus, the porous HAF CB filler in the NBR composite indicated a substantial void structure in the glass phase polymer. The solubility of hydrogen in the HAF CB-filled EPDM also indicated dual-sorption behavior, as shown in Figure 3c,d.

### 2.4. Filler Effects on H_2_ Diffusion

The hydrogen diffusivities of the neat EPDM and nine filled EPDM composites made with peroxide crosslinking were measured as a function of the applied pressure at 23 °C. The H_2_ diffusivities in neat EPDM and the EPDM composites containing fillers were apparently dependent on the exposed pressure. The pressure dependence of the diffusivity may be associated with the mean free path for hydrogen, the tortuosity caused by the impermeable filler in the rubber network, the interactions between filler and rubber, and H_2_-attractive adsorption onto the CB filler surface.

**Figure 5:**
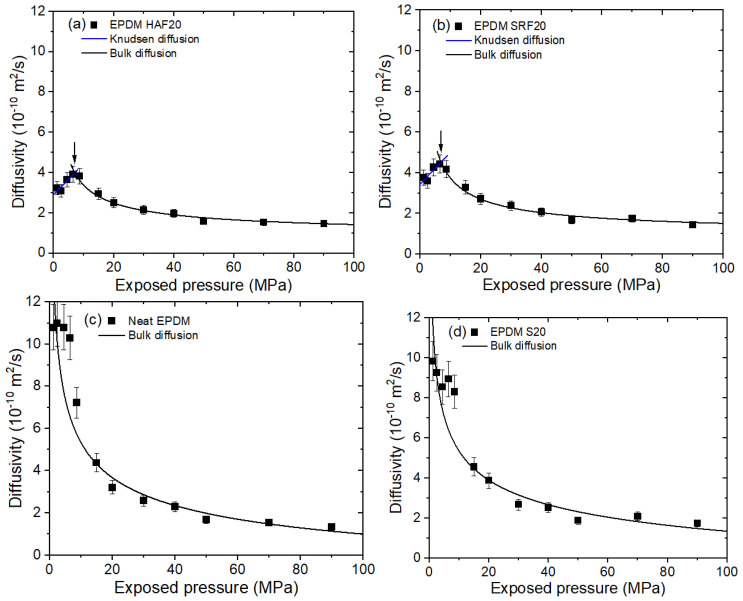
H_2_ diffusivity versus exposed pressure in (**a**) EPDM HAF20, (**b**) EPDM SRF20, (**c**) neat EPDM, and (**d**) EPDM S20. The blue lines indicate the Knudsen diffusion data fitted with Equation (4). The black lines indicate the bulk diffusion data fitted with Equation (5). The arrows in (**a**,**b**) indicate the intersection regions for Knudsen and bulk diffusion.

The representative pressure-dependent diffusion shown for EPDM HAF20 and SRF20 in Figure 5a,b, respectively, can be divided into two contributions at the peaks marked by arrows. The contributions included Knudsen diffusion at low pressure and bulk diffusion at high pressure. The pressure-dependent diffusivity was explained as a result of Knudsen diffusion below 7~10 MPa and bulk diffusion above those pressures, which was analyzed with fractal theory in other studies [44,45]. Knudsen diffusion gradually increases with increasing pressure. Knudsen diffusion at low pressures normally occurs with large mean free paths for the diffusing gas molecules or low gas densities. The Knudsen diffusivity ($D_{K,\;pm}$) in a porous medium can be written as [46]

(4) $$D_{K,pm}=\frac{\emptyset }{\tau }D_{K}=\frac{\emptyset }{\tau }\frac{d_{c}}{3}\upsilon$$

where ϕ is the pressure-dependent porosity, τ is the tortuosity caused by adding the filler, $d_{c}$ is the pore diameter, and υ is the average velocity of the gas molecules.

The bulk diffusion coefficients for neat EPDM (Figure 5c), EPDM S20 (Figure 5d), and CB-filled EPDM composites above a critical pressure of 7 MPa~10 MPa were found to be inversely proportional to pressure, which was related to the mean free path (λ) for H_2_ molecules. Bulk diffusion predominated for λ values less than the pore diameter and was found for large pores or with high-pressure gas diffusion. The bulk diffusion coefficient ($D_{B})$ can be written as [47]

(5) $$D_{B}=\frac{1}{3}\lambda \upsilon =\frac{1}{3}\frac{5}{8}\frac{\mu }{P}\sqrt{\frac{RT\pi }{2M}}\upsilon$$

where μ is the viscosity of the diffusing molecule in units of kg·m/s, and P is the applied pressure. The experimental data for the diffusivity were fitted with Equations (4) and (5), as indicated by the blue and black lines, respectively, in Figure 5. In the region of Knudsen diffusion, the diffusivity was proportional to the pressure; this was caused by an increase in the porosity in Equation (4) due to the increased pressure. The decrease in the bulk diffusion coefficient in Equation (5) was caused by decreases in λ with increasing pressure.

**Figure 6:**
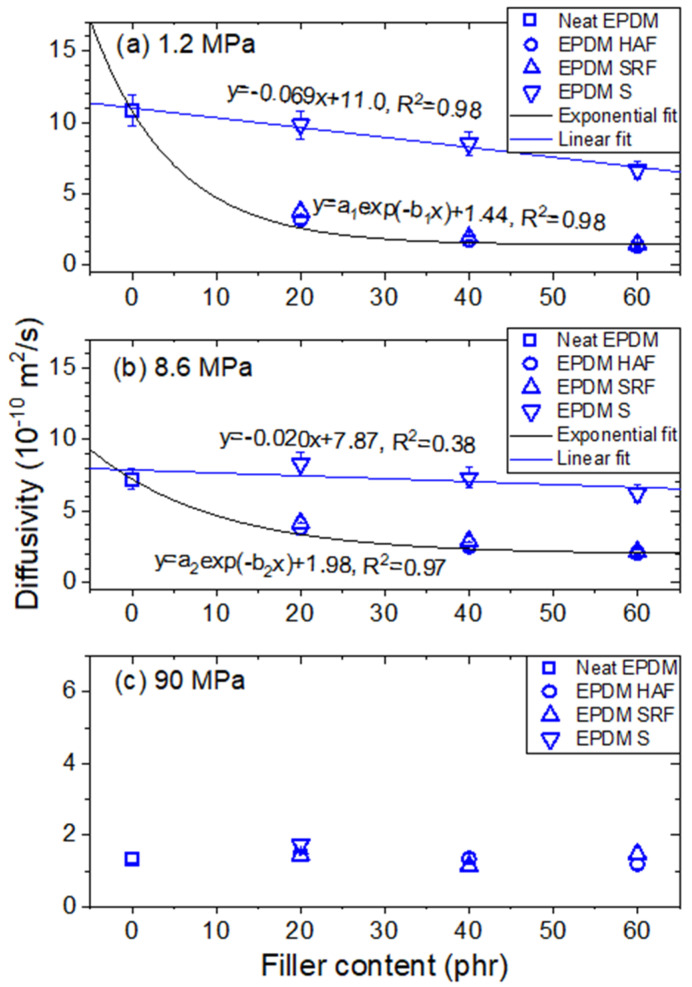
Diffusivity versus filler content for applied pressures of (**a**) 1.2 MPa, (**b**) 8.6 MPa, and (**c**) 90 MPa in peroxide-crosslinked EPDM composites blended with CB and silica. The blue lines in (**a**,**b**) are the least squares fits for the negative linear relationship between the diffusivity and filler content with their squared correlation coefficient R^2^. The black lines in (**a**,**b**) are least-squares fits with a single exponential decay relationship between the diffusivity and filler content with R^2^.

Figure 6a–c show the plots of diffusivity versus filler content for the three different pressures 1.2 MPa, 8.6 MPa, and 90 MPa, respectively. At pressures of 1.2 MPa and 8.6 MPa, the fillers extended the diffusion path due to the increased tortuosity provided by the impermeable filler, resulting in a decrease in the diffusivity. The diffusivity in the silica blended EPDM was negatively and linearly related to the filler content (i.e., (−filler content)), whereas the diffusivity in the CB-blended EPDM decreased in the form of a single exponential decay line (i.e., exp(−filler content)). At pressures up to 8.6 MPa, the decrease in the diffusivity of the CB-blended EPDM was larger than that of the silica-blended EPDM, as expected; this was possibly related to additional filler–polymer interactions or attractive H_2_ adsorption. In other words, as shown in Figure 6a,b, the diffusivity for the silica-filled EPDM was responsible for the increased tortuosity of the diffusion path introduced by the filler. However, the diffusivity of the CB-filled EPDM was attributed to polymer–filler interactions, H_2_ adsorption, and increased tortuosity. From the linear and exponential relationships seen at pressures up to 8.6 MPa, we predicted the diffusivities for different filler contents in the EPDM composites without any further diffusion measurements.

However, with increasing pressures up to 90 MPa, the effect of filler on diffusion was reduced. Thus, the diffusivities for all specimens were almost constant and converged at approximately 1.5 × 10^−10^ m^2^/s. In the pressure region of bulk diffusion, the decrease in mean free path of H_2_ molecules at 90 MPa could be a predominant factor in diffusivity behavior rather than filler content or filler species.

### 2.5. Correlations of Permeation with Physical/Mechanical Properties

**Figure 7:**
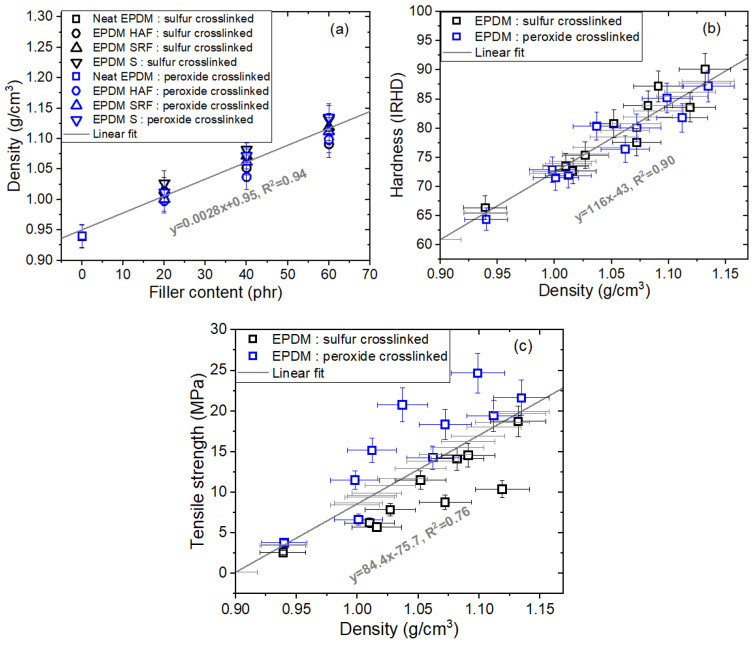
Linear correlations between (**a**) density and filler content, (**b**) hardness and density, and (**c**) tensile strength and density for EPDM composites blended with CB and silica. The data for neat EPDM are included in the linear fit to show the consistency with the fits for the filled blended EPDM composites. The black lines were fitted with the positive linear relationship. R^2^ is the squared correlation coefficient indicating the fit between the data and the linear equation.

Figure 7a depicts the density variations versus filler content for twenty EPDM composites crosslinked with sulfur and peroxide. The density increased linearly with increasing filler content and exhibited a squared correlation coefficient R^2^ = 0.94. Similarly, the linear correlation between hardness and density is represented in Figure 7b with R^2^ = 0.90. Although divergence (R^2^ = 0.76) with the data occurred, the linear correlation between tensile strength and density is also shown in Figure 7c. These three relationships indicated that their properties are linearly inter-correlated with each other. From the three linear relationships, the physical and mechanical properties of EPDM composites crosslinked with sulfur and peroxide can be easily predicted from the contents of either CB or silica fillers.

The permeability *P* was obtained by multiplying the solubility *S* by the diffusion coefficient *D*, i.e., *P* = *SD*. The filler-dependent permeability characteristics observed in previous work were very similar to those for diffusivity at 1.2 MPa and 8.6 MPa (Figure 6a,b), implying that permeability was mainly affected by the diffusivity rather than the solubility.

**Figure 8:**
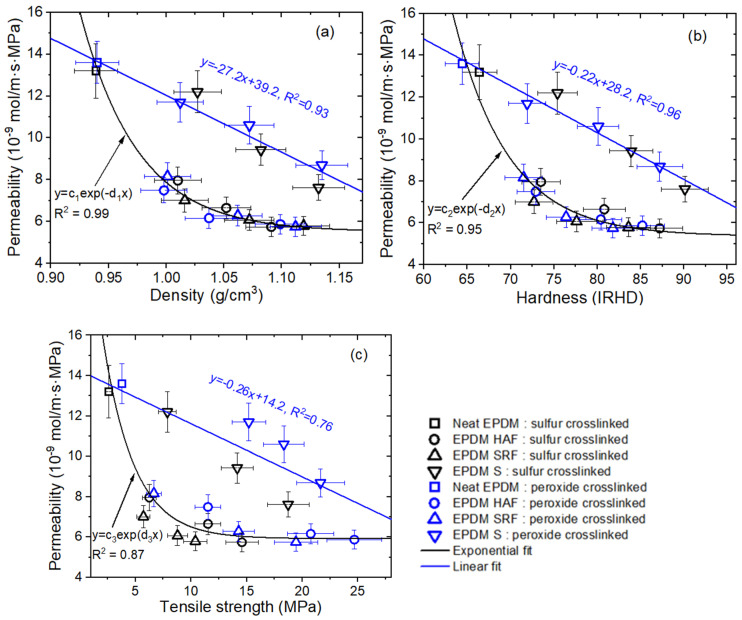
Correlations between permeability and (**a**) density, (**b**) hardness, and (**c**) tensile strength for EPDM composites blended with CB and silica. The data for neat EPDM were included to show consistency with the fits for filled blended EPDM composites. The blue lines indicate negative linear fits, and the R^2^ values are indicated. The black lines indicate fits with an exponential decay, and the R^2^ values are indicated.

Figure 8a–c show the permeability variations with density, hardness, and tensile strength, respectively, for neat EPDM and the blended EPDM polymer composites crosslinked with sulfur and peroxide.

As shown in Figure 8a, the negative linear relationship (*P* = −density) between permeability and density for silica-filled EPDM composites indicated a smooth decrease in permeation with increasing density and without any other interactions or the introduction of additional parameters. However, the effect of density on the permeability for CB-blended EPDM composites decreased exponentially with filler content, i.e., *P* = ~exp(−density). The magnitudes of the effects for CB-blended EPDM composites were larger than those for the silica-blended EPDM composites. This indicated the operation of an additional effect, i.e., strong polymer-filler interactions or attractive adsorption of H_2_ at the CB interface for CB-blended EPDM composites was included as a cause of the permeability behavior, as already shown by the pressure-dependent diffusivity seen at 1.2 MP and 8.6 MPa.

The plots of permeability versus hardness in Figure 8b and of permeability versus tensile strength in Figure 8c were very similar in form to the plots for permeability versus density in Figure 8a. Three trends may have originated from the same source. The relationships of permeation with physical and mechanical properties provide an opportunity to predict the H_2_ permeation characteristics of compounded EPDM candidates used in high-pressure seals at HRSs and HFCVs.

Moreover, to interpret the correlations between permeability and physical/mechanical properties for CB-filled EPDM composites, we must introduce the concept of free volume. This is defined as the volume of the total mass that is not occupied by the polymer chains themselves; hence, diffusing molecules could be located there. This could be due to the gaps or pores present between polymer chains. The physical/chemical parameters and density of the polymer could significantly influence the free volume of the polymer membrane.

According to free volume theory [22,26,48,49], the diffusivity and permeability are written as the fractional free volume (*FFV* = *V*_f_/*V*_SP_):

(6) $$D=a_{i}\cdot \exp\left(\frac{-b_{i}}{FFV}\right)$$

(7) $$P=c_{i}\cdot \exp\left(\frac{-d_{i}}{FFV}\right),$$

with specific free volume (*V_f_*). *V*_SP_ (cm^3^/g) is the specific volume of a polymer, equal to 1/*ρ*. *ρ* is the polymer density (g/cm^3^), and *a_i_*, *b_i_*, *c_i_,* and *d_i_* are the parameters of the given penetrant/polymer couple. In Equations (6) and (7), the diffusivity and permeability decrease exponentially with the decreasing free volume of the polymer, which negatively affects the permeation parameters.

The exponential decrease seen for the diffusivity with increasing CB filler content, as shown in Figure 6a,b, is identical to the decreases in permeability resulting from physical/mechanical properties, as shown in Figure 8a–c. According to Equations (6) and (7), the exponential behaviors imply that the CB filler content caused increases in the density, hardness, and tensile strength in the EPDM composite, leading to a decrease in the FFV, i.e., 1/FFV~density. Thus, the exponential decreases in diffusivity and permeation with density observed for the blended EPDM composites are associated with the decrease in free volume caused by adding the CB filler.

## 3. Materials and Methods

### 3.1. Sample Composition

Table 3 shows the formulations of the EPDM composites with CB and silica fillers, including one neat EPDM without filler, six specimens with CB fillers, and three specimens with silica fillers. The CB filler has two types: a high-abrasion furnace (HAF) CB and a semi-reinforcing furnace (SRF) CB, with particle sizes of 32 and 65 nm, respectively, and specific surface areas of 76 and 30 m^2^/g, respectively. The specific surface area of the silica used was 175 m^2^/g. The EPDM composites filled with fillers were designated EPDM HAFa, EPDM SRFb, and EPDM Sc, where a, b, and c denote the parts per hundred for the contents in the rubber (phr) of HAF, SRF, and silica, respectively. For example, EPDM HAF20 indicates EPDM filled with HAF CB at 20 phr. DCP (dicumyl peroxide) indicates that 1.5 phr was used as the crosslinking content. The EPDM polymers were compounded as described in the literature [50].

### 3.2. Exposure to H_2_ Gas

The conditions for exposure and purging in the high-pressure chamber used in this work have been described elsewhere [20]. Cylindrical polymer specimens with diameters of ~13 mm and thicknesses of ~3 mm were exposed to H_2_ gas for more than 24 h at pressures ranging from 1.2 to 90 MPa. After exposure to the high-pressure H_2_, the chamber valve was opened to emit the H_2_ gas.

## 4. Conclusions

We investigated the H_2_ permeation of peroxide-crosslinked EPDM composites using a volumetric analysis technique and a dedicated diffusion analysis program. The results are summarized below.

The pressure-dependent hydrogen uptake for pristine EPDM and silica-filled EPDM composites showed a single-sorption mechanism obeying Henry’s law for absorption in a polymer network. The contribution from the silica filler was negligible. However, the hydrogen uptake for the CB-filled EPDM composites presented a dual-sorption mechanism obeying Henry’s law and Langmuir’s law. The hydrogen uptake for the CB-blended EPDM was contributed by absorption in the polymer network and by adsorption in the CB filler.

The diffusivity for CB-filled EPDM composites consisted of contributions from Knudsen diffusion and bulk diffusion. The increases in the Knudsen diffusion coefficient observed below ∼10 MPa were attributed to an increase in the porosity with pressure. The decrease in the bulk diffusivity above 10 MPa was related to a decrease in the mean free path with increasing pressure. However, the only bulk diffusivities observed for neat EPDM and EPDM S20 were inversely proportional to the applied pressure.

The diffusivities for all of the EPDMs investigated were dependent on the filler type. The decrease in diffusivity for the silica-filled EPDM compared with that for pristine EPDM below 8.6 MPa was responsible for the increase in the H_2_ path tortuosity caused by introducing the filler. However, the decreases in diffusivity for the CB-filled EPDM below 8.6 MPa were caused by the polymer–filler interactions, H_2_ adsorption, and tortuosity resulting from the filler.

The silica-filled EPDMs showed negative linear correlations between diffusion and filler content, whereas the CB-filled EPDMs revealed single exponential relationships between diffusion and filler content. These relationships were very similar to the correlations between permeability and the density, hardness, and tensile strength of the EPDM composites. From these relationships, we could predict the hydrogen permeabilities of EPDM composites proposed for use as seal materials under high pressure in hydrogen infrastructure.

The single exponential decreases in diffusivity with increasing filler content and in permeation with the physical/mechanical properties for CB-filled EPDMs are responsible for the decreases in fractional free volume; these were caused by increases in the densities of the EPDM composites investigated herein, regardless of the crosslinking agents (sulfur or peroxide).

## Figures and Tables

**Table 1:** Dispersion degree of peroxide-crosslinked EPDM rubber composites blended with HAF CB and SRF CB fillers.

Composites	EPDM HAF20	EPDM HAF40	EPDM HAF60	EPDM SRF20	EPDM SRF40	EPDM SRF60
Dispersion (%)	88.3 ± 2.0	97.3 ± 0.7	97.4 ± 0.2	90.2 ± 0.4	97.4 ± 0.8	98.7 ± 0.2

**Table 2:** Fitting results with the sorption model for neat EPDM and the EPDM polymer filled with HAF CB, SRF CB, and silica fillers, according to Equation (3).

Composites	k	a	b	R^2^	Langmuir Contribution * (%)
Neat EPDM	26.2	0	0	0.99	0
EPDM HAF20	17.5	1163	0.0232	0.99	21
EPDM HAF40	16.0	2210	0.0193	0.99	35
EPDM HAF60	2.52	610	0.1941	0.95	81
EPDM SRF20	19.1	775	0.0328	0.99	18
EPDM SRF40	20.1	911	0.0374	0.99	20
EPDM SRF60	15.9	1982	0.0237	0.99	39
EPDM S20	24.5	0	0	0.99	0

* The Langmuir contribution was obtained from the total hydrogen uptake, which was the sum of the Henry and Langmuir uptake contributions.

**Table 3:** Chemical compositions of peroxide-crosslinked EPDM polymer composites blended with HAF CB, SRF CB, and silica fillers.

Composites	EPDM	ZnO	St/A	HAF N330	SRF N774	Silica S-175	Si-69	PEG	DCP	TAC
EPDM Neat	100	3.0	1.0						1.5	1.0
EPDM HAF20	100	3.0	1.0	20					1.5	1.0
EPDM HAF40	100	3.0	1.0	40					1.5	1.0
EPDM HAF60	100	3.0	1.0	60					1.5	1.0
EPDM SRF20	100	3.0	1.0		20				1.5	1.0
EPDM SRF40	100	3.0	1.0		40				1.5	1.0
EPDM SRF60	100	3.0	1.0		60				1.5	1.0
EPDM S20	100	3.0	1.0			20	1.6	0.8	1.5	1.0
EPDM S40	100	3.0	1.0			40	3.2	1.6	1.5	1.0
EPDM S60	100	3.0	1.0			60	4.8	2.4	1.5	1.0

St/A: Stearic acid; DCP: Dicumyl peroxide (crosslinking agent); TAC: Triallyl cyanurate; Si-69: Silane coupling agent; PEG: Polyethylene glycol.

## Data Availability

The data used to support the findings of this study are available from the corresponding author upon request.
